# Family Support towards Resilience in Adolescents with Type I Diabetes: A Preliminary Study in Indonesia

**DOI:** 10.31372/20190402.1028

**Published:** 2019

**Authors:** Nur Agustini, Nani Nurhaeni, Hening Pujasari, Elni Abidin, Ayu Widya Lestari, Amy Kurniawati

**Affiliations:** a Universitas Indonesia, Indonesia; b Pangkal Pinang Nursing Academy, Indonesia

**Keywords:** adolescents, diabetes, family, resilience, support, youth

## Abstract

The prevalence of diabetes has increased in adolescents. Diabetic adolescents need ongoing support from their families, and the family plays an important role in the management of the disorder. This study aimed to identify the relationship between family support and resilience in adolescents with Type 1 Diabetes Mellitus (T1DM). Quantitative data analysis was conducted using simple logistic regression. Meanwhile, qualitative data were analyzed using content analysis. The results show that the median duration of a T1DM diagnosis was 4 years, which was dominated by early adolescents in the study group (41.9%); the mean resilience score was 67.95 (range 0–100) with 53.5% of the participants had low resilience, and 55.8% of the respondents received a high level of family support. Logistic regression test results indicate that significant family support is associated with resilience.

## Introduction

Type 1 Diabetes Mellitus (T1DM) is one of the chronic diseases that until now has not been cured (Zóka et al., 2013). It is a chronic disease with impaired carbohydrate, fat, and protein metabolism which causes an insulin deficiency due to autoimmune destruction of pancreatic beta cells (Craig et al., 2014). T1DM incidents vary greatly among countries and within each country. In some countries, cases of T1DM are 5–10% of all cases of diabetes, the highest incidence is in Finland at 43/100,000 and the lowest incidence is in Japan at 1.5–2/100,000 for ages less than 15 years (Craig et al., 2014).

In Indonesia, according to the Endocrinology Children’s Coordination Unit (UKK) of the Indonesian Doctors Association (PP IDAI) Board in 2011, the number of people with T1DM in Indonesia was 3.1 per 100,000 inhabitants (Himawan, Pulungan, Tridjaja, & Batubara, 2012), and based on the T1DM national registration data from PP IDAI until 2014, it amounted to 1,021 cases (Tridjaja et al., 2015).

The American Diabetes Association (ADA) stated that early intervention for adolescents with T1DM is necessary. If the early intervention is absent, teenagers with T1DM may suffer *double diabetes*; they will suffer from both Type I Diabetes Mellitus (T1DM) and Type 2 Diabetes Mellitus (T2DM) at once due to obesity problems. In addition, the delay of intervention for diabetic teenagers can also affect short-term as well as long-term complications (ADA, 2017). Moreover, uncontrolled T1DM can lead to high rates of hospitalization causing the absence of teenagers from school and the absence of parents, as the care taker, from the workplace, triggering domestic conflicts, and also increasing morbidity and mortality (Pérez-Marín, Gómez-Rico, & Montoya-Castilla, 2015).

Dealing with T1DM, diabetic teenagers must build self-resilience in order to survive with the condition. Self-resilience, as explained by Lo et al. (2016), is an important factor so that teenage self-care for T1DM can be done consistently; thus, adolescents can adapt well to their health. Lo’s research (Scoloveno, 2015) found that resilience was positively directly related to being healthy and healthy lifestyles of adolescents aged 15–17 in New Jersey.

According to Cassarino-Perez and Dalbosco Dell’Aglio (2015), several factors influencing the level of resilience are coping strategies, family support, social skills, and optimism. Individuals with low resilience are associated with high levels of distress, poor quality of life, uncontrolled blood sugar, and non-adaptive coping mechanism (Yi-Frazier et al., 2015). However, adolescents with high resilience have positive coping strategies, good family support, good social skills, and high optimism demonstrating good metabolic control.

In this case, parents are responsible for supporting their children since family ongoing support is one of the protective factors that can increase adolescent resilience along with diet and physical exercise (Malerbi, Negrato, Gomes, & Brazilian Type 1 Diabetes Study Group (BrazDiab1SG), 2012). The support may be in a form of daily routine care, such as helping prepare food, taking medication; or even emotional support, such as being empathetic, being a good listener, and giving attention and advice if needed (Cassarino-Perez & Dalbosco Dell’Aglio, 2015). Therefore, this study aimed to identify the relationship between family support and resilience in adolescents with Type 1 Diabetes Mellitus (T1DM)

## Methods

The study design applies cross-sectional with mix method approach. The population in this study are all adolescents aged 10–20 (Keyle & Carman, 2017) with T1DM in Indonesia who joined the community of IKADAR^1^ (Family Association of Diabetic Child and Adolescent) and the WhatsApp Group until September 2017 consisting of 70 participants. Data collection began in July and ended on October 3, 2017. The reason behind retrieving respondent data from IKADAR is because T1DM cases in Indonesia have not been well-recorded. The Indonesian Pediatric Society (IDAI) documented that there were 1125 T1DM patients in 2017; however, after being confirmed, there were many patients’ addresses that had not been found. As a result, taking data from IKADAR as T1DM parents’ community was selected.

There were 43 respondents who participated in the study, based on the questionnaires sent by mail and email that were returned and sorted. The respondents were T1DM adolescents with appropriate inclusion criteria (minimum 6 months had been diagnosed with T1DM and not being admitted to hospitals). The study variables were time, old diagnosis, family support, and resilience. The measuring instrument used was a resilient measure of the Indonesian version of the Connor-Davidson Resilience Scale (CD-RISC) (*r* = 0.903) and the Hensarling Diabetes Family Support Scale (HDFSS) version of Indonesia (*r* = 0.96). In-depth interviews were conducted on two participants selected purposively based on the extreme scores: one with the highest resilience and level of family support, male, aged 17 years old, and has been diagnosed with T1DM for nine years; and, the other one with the lowest resilience and level of family support, female, aged 16 years old, and has been diagnosed with T1DM for three years.

## Results

Table 1 and Table 2 indicate that the majority of the respondents are early adolescents aged 10–13 years old (41.9%) who have been diagnosed with T1DM for four years (Minimum 1–Maximum 12 years). The average resilience score is 67.95 (range 0–00), and the majority have low resilience (53.5%). Family support gained is mostly high (55.8%).

**Table 1:** The Average Score of Duration of Diagnosis and Resilience Score in the IKADAR Indonesia Community, October 2017 (N = 43)

Variable	Mean	SD	Median	(Min-Max)	Normality (Shapiro Wilk)
Duration of diagnosis (year)	4.356	2.86	4	(1–12)	*p* = 0.003
Resilience (score 0–100)	67.95	9.89	69	(49–91)	*p* = 0.717

**Table 2:** Frequency Distribution based on Age, Resilience Level, and Family Support in IKADAR Indonesia Community, October 2017 (N = 43)

Variable	*n*	Percentage (%)
**Age**		
Late adolescence (age 17–20)	10	23.3
Middle adolescence (age 14–16)	15	34.9
Early adolescence (age 10–13)	18	41.9
**Resilience**		
High	20	46.5
Low	23	53.5
**Family support**		
High	24	55.8
Low	19	44.2

To determine the variables that affect resilience, a multivariate test with logistic regression was performed. The test results are shown in Table 3 which explains that based on the logistic regression test, family support is significantly associated with resilience (OR = 4.67; *p* < 0.05), suggesting that family support is able to increase adolescent resilience 4.67 times. Based on the family support questionnaire, of four types of support (information, emotional, award, and instrumental support), only information support affects resilience (OR = 2.21; *p* < 0.05).

**Table 3:** Variables influencing Resilience T1DM Adolescents in IKADAR Community, October 2017 (N = 43)

		Resilience								
		High *n* (%)		Low *n* (%)		Total		*p* value		OR (CI 95%)
**Age**										
Late adolescence (age 17–20)		6 (30)		4 (17.4)		10				2.362 (0.334–16.726)
Middle adolescence (age 14–16)		6 (30)		9 (39.1)		15		0.601		
Early adolescence (age 10–13)		8 (44.4)		10 (43.5)		18				
**Duration of diagnosis**		20 (46.5)		23 (53.5)		43		0.087		0.616 (0.420–1.903)
**Family support**										
High		15 (75)		9 (39.1)		24		0.018		4.673 (1.784–26.360)
Low		5 (25)		14 (60.9)		19				

In-depth interviews were conducted with two respondents with the extreme scores: one with the highest resilience and level of family support, male, aged 17 years old, and has been diagnosed with T1DM for nine years (respondent 1); and, the other one with the lowest resilience and level of family support, female, aged 16 years old, and has been diagnosed with T1DM for three years (respondent 2).

The results of the content analysis identified various themes, such as boredom, in addition to feeling overwhelmed and burdened by the need for daily treatment (i.e., blood sugar and insulin injections), as illustrated below:

“So, how I feel is like I feel burdened already by the need to have injections every day. I have to get shots over and over again, like so many times, four times a day. I’m just sick of always remembering the schedule. Sometimes, I just forget it. Sometimes, yeah, I just feel tired.” (Respondent 1)

“Sometimes I feel burdened too, like being overwhelmed that I have to check my blood sugar level every day, like feeling wary and lazy. Honestly, it is really tiring. I don’t like it, but I have no choice, so that it’s more tiring, more complicated.” (Respondent 2)

One of the respondents occasionally expressed non-compliance with prescribed treatment and care, as illustrated below:

“While hanging out with friends, sometimes when I get my head straight, I only watch my friends having their meals. But, when I act carelessly, I just grab anything my friends have although I know it’s bad for me (laughter). I don’t care how the meal will affect my glucose level and I don’t think of the carbs anymore.” (Respondent 2)

In addition to the negative responses about having T1DM, there were also positive responses, as shown below:

“I have diabetes. This means having to deal with injections. We believe that life is given by God and that disease is a trial for our faith meaning that I should have got through this. Insha Allah (if God wills), the sickness is a way to cleanse myself from my sin. Yes, I can get through this sincerely.” (Respondent 1)

“I think that people assume that diabetes is such a disease. Actually, we are just like ordinary people. The difference is we must check out our blood sugar level, get shots, and set the diet. We do normal activities. I still can eat normally. That is important (laughter).” (Respondent 1)

“Stay positive, do not give up. When you have diabetes, you can be stressful. But for me, I’d keep motivated. As my teachers say, I’m a highly motivated person. That is why I am always in high spirits though I need to get shots every day. Alhamdulillah (Thank God) I still can earn achievement from school, and never fails in class.” (Respondent 2)

The respondents stated that the support of others, such as peers, teachers, and of course parents, was very helpful in motivating them. As noted by one of the respondents:

“My mom practiced checking my blood sugar, just like me. After one week, she was already skillful. Finally, I was allowed to go home (from hospital).” (Respondent 1)

“In the hospital, my mom learned about the syringe injection (injection schedule and diet).” (Respondent 2)

“He (close friend) supports me, reminds me not to get too tired and not to miss insulin injections. He reminds me to check my blood sugar, and not to eat too much. He always reminds me.” (Respondent 1)

## Discussion

The study identified that the average resilience score among adolescents with T1DM was 67.95 and adolescent with low resilience became the majority (53.5%) with score below 67.95. This score was lower than with an average score of diabetic patients in the USA (83.1), in Taiwan (74.9), and in Brazil with an average score of 79.8 (Davidson & Connor, 2017).

By age group, it was found that late adolescents groups had higher resilience compared with early adolescents and middle adolescents. This finding is in line with the research (Yi-Frazier et al., 2010) which explains that the older people are, the better resilience they gain.

Adolescent resilience scores can be used as a reference to determine the necessary nursing actions since low-resilience individuals will experience more distress, poorer quality of life, and less control of blood glucose levels in their body (Yi-Frazier et al., 2015). This opinion is reinforced by a notion (Wu et al., 2013) stating that resilience is a factor of success in adapting to an effective response toward environmental changes and the effects of sustained stress. A study found that direct and positive resilience was associated with health, positive expectations, and healthy lifestyles (*p* < 0.01) in adolescents aged 15–17 (Scoloveno, 2015). Moreover, resilience causes individuals to have the power to adapt, withstand stress, and potentially increase the ability to deal with adversity (Edward, 2013).

Bivariate test results showed that family support were associated with resilience (*p* < 0.05). Adolescents with high resilience and positive support from schools are significantly associated with their ability to perform diabetes care independently. This can be seen from the average hemoglobin A1c (HbA1c) levels during the last 2–3 months and the good quality of life (Lo et al., 2016). The result of this study indicates that high family support is predicted to increase resilience 4.67 times compared with lack of family support.

The existence of family support is expected to increase resilience and will ultimately have an impact on increasing the ability of adolescents in performing diabetes treatment independently. Another study also found that family support was significantly associated with adherence to diabetes therapy in diabetic adolescents (Pereira, Berg-Cross, Almeida, & Machado, 2008). These findings highlight the importance of focusing nursing care on the strengths of individuals and families rather than just focus on meeting the needs or the problems of a patient. The power of the family has the potential to facilitate the transformation and improvement of family capacity in facing sudden or prolonged changes (West, Usher, & Foster, 2011).

The quantitative data showed that family support was associated with the adolescent resilience, particularly in the role of providing information. This finding was supported by qualitative data. Two participants asserted that their mothers were strongly eager to be able to help their children performing home therapy. The mothers did learn how to give insulin injection, checking blood sugar, and also managing the diet.

Information support becomes important in the management of diabetes for adolescents. Family involvement as a complement to the implementation of information delivery can increase the role of adolescents in self-care. Positive support from the family can reduce the stress of children and teenagers due to diabetes, so that they can improve the implementation of better self-care (Naranjo, Mulvaney, McGrath, Garnero, & Hood, 2014).

Family support is one of the protective factors that can increase adolescent resilience. Theory of Resilience in Illness Model (RIM) explained that resilience is a multidimensional concept with two factors that play a role: factors that can increase resilience (protective factor) and can decrease resilience (risk factor). Some factors identified as protective factors are family (family acceptance and closeness, ability to communicate effectively, family strength), social integration (peer support and health care), positive expectations, spirituality, and courageous coping. While the factors identified as risk factors are distress and defensive coping (Haase, Kintner, Monahan, & Robb, 2014).

The role of supporting systems provided by parents or families is helping adolescents creating positive coping strategies in overcoming physical and psychosocial problems. This is in line with some research literature declaring that the support of family and surrounding is very helpful to adolescents in achieving physiological and psychosocial adaptation. The support will enhance positive coping strategy in adolescents so as helping to achieve good metabolic control through adherence in care and improving the quality of life of adolescents (Pérez-Marín et al., 2015).

## Conclusion

This study shows that T1DM adolescents need support from families to improve self-resilience in order to be always motivated to take care of themselves for rest of their lives. In addition, the significant family support is dealing with information that can increase the knowledge and the ability of the family in taking care of their children with T1DM at home. In this case, nurses as agents of change should always pay attention to the patient’s biopsychosocial needs and should always involve family in the provision of care by applying family centered care.

## Declaration of Conflicting Interests

The authors declared no potential conflicts of interest with respect to the research, authorship, and/or publication of this article.

## Funding

This study was funded by Hibah Riset Madya FIK UI, Year 2017.

## Endnotes

1 IKADAR is Indonesia’s diabetes community to connect and support children and teenage with diabetes. They can be found via Instagram @ikadarindonesia and Facebook Public Group *IKADAR INDONESIA* https://www.facebook.com/groups/119499111452412/.
